# Iron–induced, sex‐specific alterations of mitochondrial bioenergetic function and increased amyloid beta plaque size in 5xFAD mice

**DOI:** 10.1002/alz.090717

**Published:** 2025-01-03

**Authors:** Wilson Peng, Mark Osabutey, Chidozie N Okoye, Kaitlin Chung, Lee Trojanczyk, Linh Le, Nada Ahmed Selim, Alex S Milliken, Ali Al‐Qazzaz, Ania K Majewska, Andrew P Wojtovich, Robert T Dirksen, M. Kerry O'Banion, John O Onukwufor

**Affiliations:** ^1^ University of Rochester School of Medicine & Dentistry, Rochester, NY USA

## Abstract

**Background:**

Alzheimer’s disease (AD) is a progressive neurodegenerative disease characterized by neuronal dysfunction leading to decreased memory and cognitive function. AD research has largely focused on the potential pathogenic role of two disease hallmarks: amyloid beta and phosphorylated tau. However, pharmacological interventions targeting these disease hallmarks have met with limited clinical trial success. Thus, other variables may initiate disease etiology prior or contribute to the development of tau and amyloid pathologies. Iron is essential for brain function where its tight regulation aids in neurotransmitter biosynthesis and energy homeostasis. AD patients exhibit high levels of iron in their brain relative to age‐matched, non‐AD patients. However, the potential role of high brain iron levels in AD pathogenesis is unclear.

**Method:**

Eight‐week‐old male and female C57BL/6 and 5xFAD mice were exposed to standard chow or iron 8 g/kg chow for 8 weeks. Half of the brain from each mouse was used to assess mitochondrial bioenergetic function, with the remaining half quantified for amyloid beta plaque number and size.

**Result:**

Iron treatment significantly enhanced state 3 (maximum) respiration in male 5xFAD mice, but not in female 5xFAD mice. In contrast, iron treatment increased state 4 respiration in both male and female 5xFAD mice, though the effect in female mice was greater. As a result, mitochondrial respiratory control ratio was greatly impaired in both sexes. Furthermore, iron treatment significantly increased average plaque size in female 5xFAD mice with no effect on male 5xFAD mice, while the total number of plaques were not different in either sex.

**Conclusion:**

Our data suggest that iron treatment induced sex‐specific alterations of mitochondrial bioenergetic function and amyloid beta plaque size in 5xFAD mice. Future studies will assess the underlying molecular mechanisms driving sex‐specific differences in iron‐induced AD pathology.

